# *Tiliacora triandra*, an Anti-Intoxication Plant, Improves Memory Impairment, Neurodegeneration, Cholinergic Function, and Oxidative Stress in Hippocampus of Ethanol Dependence Rats

**DOI:** 10.1155/2015/918426

**Published:** 2015-06-09

**Authors:** Nattaporn Phunchago, Jintanaporn Wattanathorn, Kowit Chaisiwamongkol

**Affiliations:** ^1^Department of Physiology (Neuroscience Program) and Graduate School, Faculty of Medicine, Khon Kaen University, Khon Kaen 40002, Thailand; ^2^Integrative Complementary Alternative Medicine Research and Development Center, Khon Kaen University, Khon Kaen 40002, Thailand; ^3^Department of Physiology, Faculty of Medicine, Khon Kaen University, Khon Kaen 40002, Thailand; ^4^Department of Anatomy, Faculty of Medicine, Khon Kaen University, Khon Kaen 40002, Thailand

## Abstract

Oxidative stress plays an important role in brain dysfunctions induced by alcohol. Since less therapeutic agent against cognitive deficit and brain damage induced by chronic alcohol consumption is less available, we aimed to assess the effect of *Tiliacora triandra* extract, a plant possessing antioxidant activity, on memory impairment, neuron density, cholinergic function, and oxidative stress in hippocampus of alcoholic rats. Male Wistar rats were induced ethanol dependence condition by semivoluntary intake of alcohol for 15 weeks. Alcoholic rats were orally given *T. triandra* at doses of 100, 200, and 400 mg·kg^−1^BW for 14 days. Memory assessment was performed every 7 days while neuron density, activities of AChE, SOD, CAT, and GSH-Px and, MDA level in hippocampus were assessed at the end of study. Interestingly, the extract mitigated the increased escape latency, AChE and MDA level. The extract also mitigated the decreased retention time, SOD, CAT, and GSH-Px activities, and neurons density in hippocampus induced by alcohol. These data suggested that the extract improved memory deficit in alcoholic rats partly via the decreased oxidative stress and the suppression of AChE. Therefore, *T. triandra* is the potential reagent for treating brain dysfunction induced by alcohol. However, further researches are necessary to understand the detail mechanism and possible active ingredient.

## 1. Introduction

Alcohol (ethanol) consumption in Thailand is dramatically increased. Data obtained from alcohol consumption collected by World Health Organization have demonstrated that Thailand is the top ethanol-consuming country in Asia and 40% of drinkers are in North-East region [1]. Chronic ethanol exposure can produce multiple and durable changes in the central nervous system. It has been reported that chronic ethanol consumption produces a significant loss of brain tissues especially in forebrain and hippocampus [2] together with the neurodegeneration of cholinergic neurons in basal forebrain [3]. Several lines of evidence from animal study have also revealed that a specific neuronal loss in the dentate gyrus, increased arborizations of the dendritic spines of the granule cells, a reduction of the number of spines of the CA3-pyramidal cells, and a reorganization of synaptic formations [4–7] are also presented. In addition, chronic ethanol consumption also induces memory impairment. This impairment has been demonstrated to relate with hippocampus degeneration and cholinergic function [8].

Accumulative lines of evidence have demonstrated that neurodegeneration induced by chronic ethanol consumption is associated with the elevation of oxidative stress [9, 10]. The elevation of oxidative stress induced by chronic ethanol consumption is reported to occur both via the increased free radical formation and via the decreased antioxidant enzyme activities [11]. In addition, these changes can be mitigated by the substances possessing antioxidant activity [10]. Currently, drugs which target at protecting against brain damage and memory impairment in alcoholism are less available. Therefore, the therapeutic benefit of herbal medicine has gained much attention especially in Asian countries [12].

*Tiliacora triandra* (Colebr.) Diels or Ya-nang in Thai belongs to the family of Menispermaceae. It is the native plant of Southeast Asia and widely used in the cuisines of northeast Thailand and Laos. Ya-nang is used not only as food but also as medicine in traditional folklore. According to the traditional medicine of many countries in Southeast Asia, it has been used as anti-pyretic, detoxication agent, anti-inflammation, anticancer, antibacterial, and immune modulator. In addition, it also possesses antioxidant activity [13]. Recent toxicity study has revealed that water extract of* T. triandra* leaves shows no toxicity up to 5000 mg·kg^−1^ in single administration. Moreover, no adverse effects were observed following the subchronic administration of the water extract of this plant at doses of 300, 600, and 1200 mg·kg^−1^ [14]. Based on the antioxidant effect together with the detoxification reputation of this herb and the benefit of substance possessing antioxidant activity against ethanol neurotoxicity mentioned earlier, the health benefit against neurotoxicity of* T. triandra* extract has been considered. Thus, this study was carried out to determine the effect of water extract of* T. triandra* on memory impairment, neurodegeneration, cholinergic function, and oxidative stress in hippocampus of ethanol dependence rats.

## 2. Materials and Methods

### 2.1. Experimental Animals

Adult male Wistar rats, 8 weeks old, were used as experimental animals. They were purchased from National Laboratory Animal Center, Salaya, Nakorn Pathom. All animals were acclimatized for two weeks on normal diet of rat chow, with water given ad libitum at room temperature with a 12-hour light and dark cycle before the commencement of the experiment. The weights of the animals on the first day of experiment were 180–220 g. The experiments were performed to minimize animals' suffering and the experiment protocols were approved by the Institutional Animal Care and Unit Committee Khon Kaen University, Thailand.

### 2.2. Plant Material and Extract Preparation

The aerial parts of* T. triandra* were collected from Khon Kaen province, Thailand. The plant was authenticated by Associate Professor Panee Siri-sa-ard, Faculty of Pharmacy, Chiangmai University, Chiangmai, Thailand (authentication number 023160). They were cleaned dried and ground to fine powder. Then, the ground powder was boiled with distilled water at a ratio of 1 : 6 (w/v) for 5 minutes with a continuous stirring. After being left at room temperature for 24 hours, the extract was filtered using Whatman No. 1. The filtrate was evaporated under reduced pressure using a rotary evaporator [15]. The percent yield of the extract was 5.5.

### 2.3. Sample Analysis

The total phenolic compounds content was determined with the Folin-Ciocalteu reagent. Gallic acid was used as a standard and the total phenolics were expressed as mg/g gallic acid equivalents (GAE) [16].

The finger print of* T. triandra* leaves extract was carried out by using gradient high performance liquid chromatography (HPLC) system. The system consists of 515 HPLC pump and 2998 Photodiode array detector of Waters company, USA. Chromatographic separation was performed using Purospher STAR, C-18 encapped (5 *μ*m), LiChroCART 250-4.6, and HPLC-Cartridge, Sorbet Lot number HX255346 (Merk, Germany). Two mobile phases consisting of methanol and 2.5% acetic acid in deionized (DI) water were used to induce gradient elution. The injection volume was 20 *μ*L and the flow rate was 1.0 mL/min. During HPLC analysis the solvent gradient was programmed as shown in Table 1 and data analysis was performed using Empower 3.

### 2.4. Experimental Protocol

The animals were induced ethanol dependence by using a semivoluntary intermittent intake method [17]. In brief, rats were exposed to ethanol in drinking water. The ethanol concentration in drinking water was gradually increased in a stepwise fashion from 5% at a rate of 5% per week until reaching a 20% ethanol wihin 4 weeks in order to allow the animals to get used to the taste of alcohol and mimic the condition of alcohol addiction in human. Then, the ethanol concentration was raised to 30% ethanol and maintained at this concentration between the fifth and the fifteenth weeks. The hyperexcitability symptoms which reflected ethanol dependence condition including the increased response to environmental stimuli such as startle, irritability, and hypervigilance were evaluated [17]. In addition, all ethanol dependence rats used in this study showed the average ethanol concentration at 9.89 ± 0.86 mg/dL prior to the intervention. The ethanol dependence rats were selected for further study and randomly assigned to 7 groups of 6 animals each as follows.

 Group I: control group: rats in this group received no treatment.
 Group II: ethanol + vehicle: alcoholic rats were orally administered vehicle (distilled water).
 Group III: ethanol + Aricept (donepezil): ethanol dependence rats were administered donepezil via oral route at dose of 1 mg/kg^−1^BW (this group served as positive control because this drug has been used as standard drug for treating memory deficit patients) [18].
 Group IV: ethanol + vitamin C: ethanol dependence rats were administered vitamin C at dose of 250 mg/kg^−1^BW. (This group also served as positive control based on the previous findings that the substances possessing antioxidant effect exert neuroprotective effect against alcohol neurotoxicity of vitamin C [19].)
 Group V: ethanol +* T. triandra* 100: ethanol dependence rats were orally administered* T. triandra* extract at dose of 100 mg·kg^−1^BW.
 Group VI: ethanol +* T. triandra* 200: ethanol dependence rats were orally administered* T. triandra* extract at dose of 200 mg·kg^−1^BW,
 Group VII: ethanol +* T. triandra* 400: ethanol dependence rats were orally administered* T. triandra* extract at dose of 400 mg·kg^−1^BW.

All animals received the assigned treatments at a period of 14 days after the induction of ethanol dependence by a 15-week alcohol treatment. They were assessed spatial memory using Morris water maze test after the single intervention, 7 days and 14 days of treatments. At the end of experiment, they were sacrificed and hippocampi were isolated for the determination of oxidative damage markers including the level of malondialdehyde (MDA) and the activities of catalase (CAT), superoxide dismutase (SOD), and glutathione peroxidase (GSH-Px). In addition, the activity of acetylcholinesterase (AChE) in hippocampus, the crucial area for learning and memory, was also determined.

### 2.5. Determination of Spatial Memory

Spatial memory was evaluated using Morris water maze test. According to this test, a circular pool (160 cm in diameter × 60 cm height) filled with water (approximately 42 cm deep, temperature of 23-24°C) was divided into 4 quadrants. The removable platform was placed in the center on one quadrant under the water surface. The immersed platform was invisible due to the masking effect of nontoxic milk powder which covered the water surface. The animal must memorize the location of the immersed platform by forming the association memory between its location and the location of the platform via the environmental cues. The time which the animal spent to find the hidden platform and climb onto the platform was recorded as escape latency. The platform was removed 24 hr later and the animal was reexposed to the test again to evaluate the memory retention and retrieval capacity. The time which the animals spent swimming in the quadrant which the platform was previously located was recorded as the retention time [20, 21].

### 2.6. Determination of Oxidative Stress Markers

Rats were perfused with cold saline solution to get rid of the blood from the brain tissue; then, hippocampi were rapidly removed and stored at −80°C until used. To determine the oxidative stress markers, brains were prepared as homogenate and we determined the level of malondialdehyde (MDA) using the thiobarbituric acid reaction [22] whereas glutathione peroxidase (GSH-Px), catalase (CAT), and superoxide dismutase (SOD) were determined using a spectrophotometric method [23].

#### 2.6.1. Determination of Malondialdehyde (MDA) Level

Level of malondialdehyde (MDA), a relatively stable lipid peroxidation marker, was determined by using thiobarbituric acid reacting substances (TBARS) assay. MDA which occurred as the result of the breakdown of polyunsaturated fatty acid must react with thiobarbituric acid reacting substances and gives rise to the pink color product. This product could be measured at 532 nm. In this test, 1,3,3-tetra ethoxy propane (TEP) was used as the standard.

#### 2.6.2. Catalase (CAT) Assay

Catalase activity was determined spectrophotometrically by measuring the decrease in H_2_O_2_ absorbance at 490 nm. A system devoid of the substrate (hydrogen peroxide) served as the control. The difference in absorbance per unit time was expressed as the activity. One unit was defined as the amount of enzyme required to decompose 1.0 M of hydrogen peroxide per minute at pH 7.0 and 25°C.

#### 2.6.3. Superoxide Dismutase (SOD) Assay

The activity of SOD was performed using a xanthine/xanthine oxidase system for the production of superoxide radical and subsequent measurement of cytochrome c as a scavenger of the radicals. Optical density was determined using a spectrometer (UV-1601, Shimadzu) at 550 nm. A system devoid of enzyme served as control. One unit of enzyme activity was defined as the quantity of SOD required to inhibit the rate of reduction of cytochrome by 50%. SOD activity was presented as units per milligram of protein (U/mg protein).

#### 2.6.4. Glutathione Peroxidase (GSH-Px) Assay

The determination of GSH-Px was performed using t-butyl hydroperoxide as a substrate. The optical density was recorded at 340 nm. One unit of the enzyme was defined as micromole (*μ*mol) of reduced nicotinamide adenine dinucleotide phosphate (NADPH) oxidized per minute. GSH-Px activity was expressed as U/mg protein.

### 2.7. Determination of Acetylcholinesterase (AChE) Activity

Acetylcholinesterase (AChE) was analyzed based on the basis that this enzyme catalyzed the hydrolysis of acetylthiocholine (ATCh) which in turn gave rise to the formation of acetate and thiocholine. In the presence of the highly reactive 5,5′-dithiobis-(2-nitrobenzoic acid) (DTNB) ion, thiocholine generated a yellow color substance which was quantitatively monitored by spectrophotometric absorption at 405 nm [24].

### 2.8. Histological Study

#### 2.8.1. Tissue Preparation

At the end of study, all rats were exposed to transcardial perfusion. In brief, the thorax was carefully cut open, the heart was exposed, and a needle connected to the tubing from the fixative bottle was inserted into the left ventricle. The right atrium was cut open to drain out the blood and fixative. First 20–30 mL of saline was passed transcardially to flush out the blood and then perfuse with 4% paraformaldehyde in 0.1 M phosphate buffer pH 7.4. Fixation was monitored by the gradual discolorations of the tongue and eyeball. After fixation has been established, the brain specimens were further kept in the fixative containing 30% sucrose for 72 h. Regions of hippocampus were then dissected out using Paxinos stereotaxic coordinate method [25]. Serial sections of tissues containing hippocampus area were prepared using a sliding microtome at 20 *μ*m thick. Tissue samples were picked up on slides coated with a 0.01% aqueous solution poly-L-lysine.

#### 2.8.2. Cresyl Violet Staining of Brain Tissue

The sections containing hippocampus of all groups were stained with 0.5% cresyl violet. Analysis of neuronal density was performed on coronal sections of the dorsal hippocampus stained with cresyl violet and corresponding to brain sections located between 3.14 and 4.16 mm posterior to bregma [25]. The densities of living neurons in CA1, CA2, CA3, and dentate gyrus were performed using Olympus light microscope model BH-2 at 40x magnification by an observer who was blind to the treatment at time of analysis.

### 2.9. Statistical Analysis

Data were presented as mean ± standard error of mean (SEM). Statistical analysis was analyzed using one-way analysis of variance (ANOVA), followed by LSD post hoc test. Probability levels less than 0.05 were accepted as significance.

## 3. Results

### 3.1. Sample Analysis

**Figure 1:**
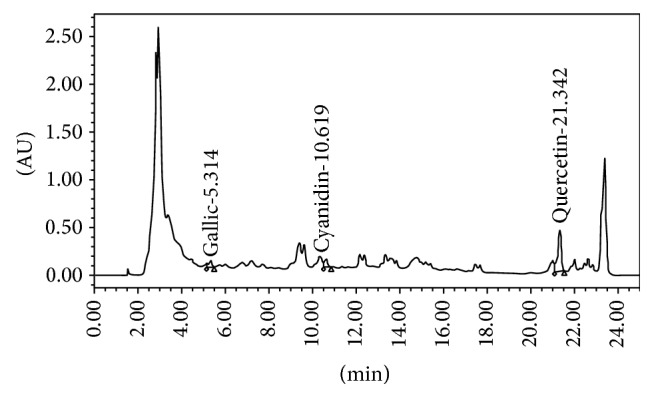
The chromatogram of water extract of* T. triandra*.

The data obtained from this study showed that the* T. triandra* leaves extract used in this study contained total phenolic compounds at concentration of 593.33 mg of gallic acid equivalent (GAE)/mg extract. The HPLC fingerprint of* T. triandra* was shown in Figure 1. We had identified gallic acid, cyanidin, and quercetin according to their retention times and spectral characteristics of their peaks compared with standard. The ultraviolet spectrum of chromatographic bands presented in the fingerprinting of the samples indicated the presence of gallic acid at a concentration of 4.81 ± 0.05 *μ*g gallic/100 mg of extract whereas the concentrations of cyanidins and quercetin were presented at the concentrations of 307.22 ± 4.74 *μ*g Cyn-3-glu/100 mg and 9028.86 ± 695.97 *μ*g QE/100 mg extract, respectively.

### 3.2. Effect of* T. triandra* Extract on Spatial Memory

**Figure 2:**
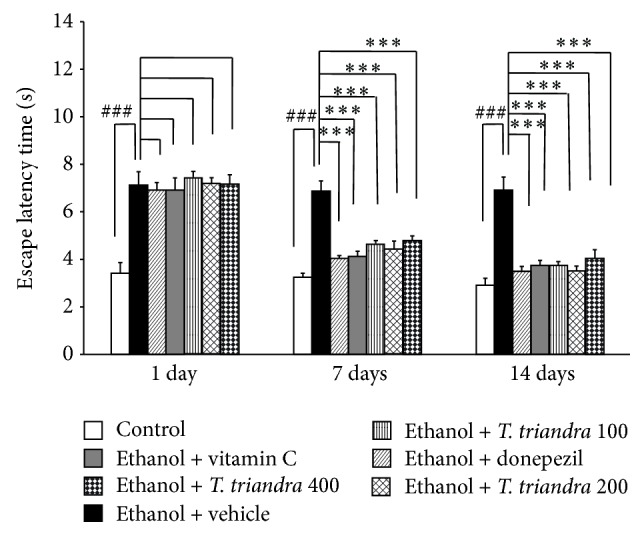
The effect of* T. triandra* on escape latency time in Morris water maze test. Data were presented as mean ± SEM, *n* = 6/group.^ ###^ *P* value < 0.001 compared with control treated group. ^∗∗∗^ *P* value < 0.001 compared with ethanol dependence treated group which received vehicle.

Figure 2 showed that ethanol dependence rats induced by 15-week alcohol consumption which received vehicle or water showed the enhanced escape latency since the first day of treatment until the end of 14-day study period (*P* value < 0.001 all, compared to control rats). Donepezil, vitamin C, and all doses of* T. triandra* failed to modulate the enhanced escape latency induced by ethanol at 1-day treatment period. However, when the treatments were prolonged to 7 and 14 days, ethanol dependence rats which received donepezil or vitamin C or* T. triandra* extract at doses of 100, 200, and 400 mg·kg^−1^BW significantly mitigated the elevation of escape latency induced by ethanol (*P* value < 0.001 all, compared with ethanol dependence rats which received vehicle).

**Figure 3:**
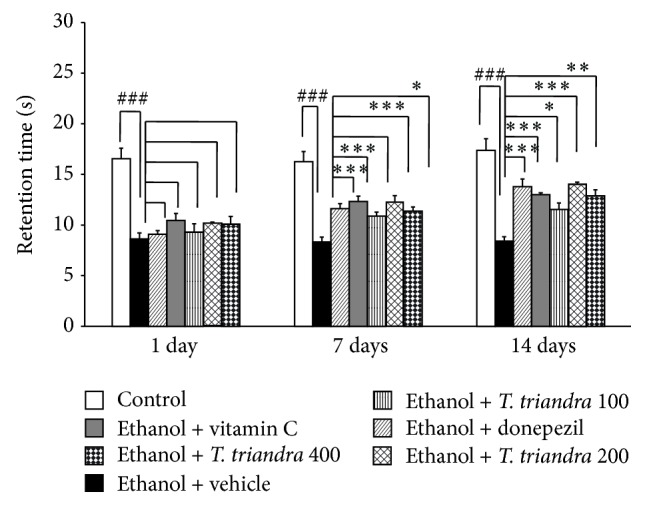
The effect of* T. triandra* on retention time in Morris water maze test. Data were presented as mean ± SEM, *n* = 6/group.^ ###^ *P* value < 0.001 compared with control treated group. ^∗^ *P* value < 0.05 compared with ethanol dependence treated group which received vehicle. ^∗∗∗^ *P* value < 0.001 compared with ethanol dependence treated group which received vehicle.

The effect of* T. triandra* on retention time in Morris water maze test was also investigated and results were shown in Figure 3. It was found that ethanol dependence rats which received vehicle showed the decreased retention time throughout the 14-day study period (*P* value < 0.001 all, compared to control rats). Ethanol dependence rats which received either donepezil or vitamin C significantly mitigated the decreased retention time induced by alcohol consumption at 7 and 14 days of treatment (*P* value < 0.001 all, compared with ethanol dependence rats which received vehicle). In addition, ethanol dependence rats which received* T. triandra* at doses of 200 and 400 mg·kg^−1^BW also showed the significant attenuation of the decreased retention time induced by ethanol consumption both at 7 (*P* value < 0.001 and 0.05, resp., compared with ethanol dependence rats which received vehicle) and 14 days of treatments (*P* value < 0.001 all, compared with ethanol dependence rats which received vehicle). However, ethanol dependence rats which received* T. triandra* at dose of 100 mg·kg^−1^BW significantly attenuated the decreased retention time induced by ethanol consumption only at 14 days of treatment (*P* value < 0.05, compared with ethanol dependence rats which received vehicle).

### 3.3. Effect of* T. triandra* on AChE Activity in Hippocampus

**Figure 4:**
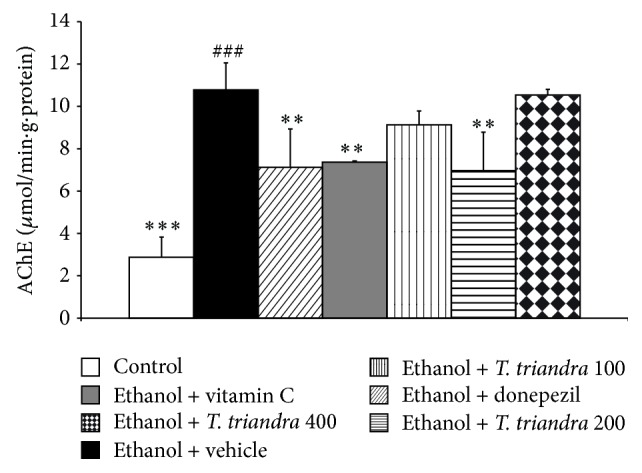
The effect of* T. triandra* on the activity of acetylcholinesterase (AChE) in hippocampus. Data were presented as mean ± SEM, *n* = 6/group ^###^ *P* value < 0.001 compared with control treated group. ^∗∗^ *P* value < 0.01 compared with ethanol dependence treated group which received vehicle. ^∗∗∗^ *P* value < 0.001 compared with ethanol dependence treated group which received vehicle.

Since cholinergic system played an important role on spatial memory, we also investigated the effect of* T. triandra* on AChE activity in this area. The results were shown in Figure 4. The significant elevation of AChE activity in hippocampus was observed in ethanol dependence rats which received vehicle (*P* value < 0.001, compared with control rats). Treatment with either donepezil or vitamin C could mitigate the elevation of AChE in hippocampus (*P* value < 0.01 all, compared with ethanol dependence rats which received vehicle). Ethanol dependence rats which received the extract at dose of 200 mg·kg^−1^BW also significantly mitigated an elevation of AChE activity induced by ethanol consumption in hippocampus (*P* value < 0.01 all, compared with ethanol dependence rats which received vehicle) while no changes were observed in ethanol dependence rats which received low and high doses of extract.

### 3.4. Effect of* T. triandra* on Neuron Density in Hippocampus

**Figure 5:**
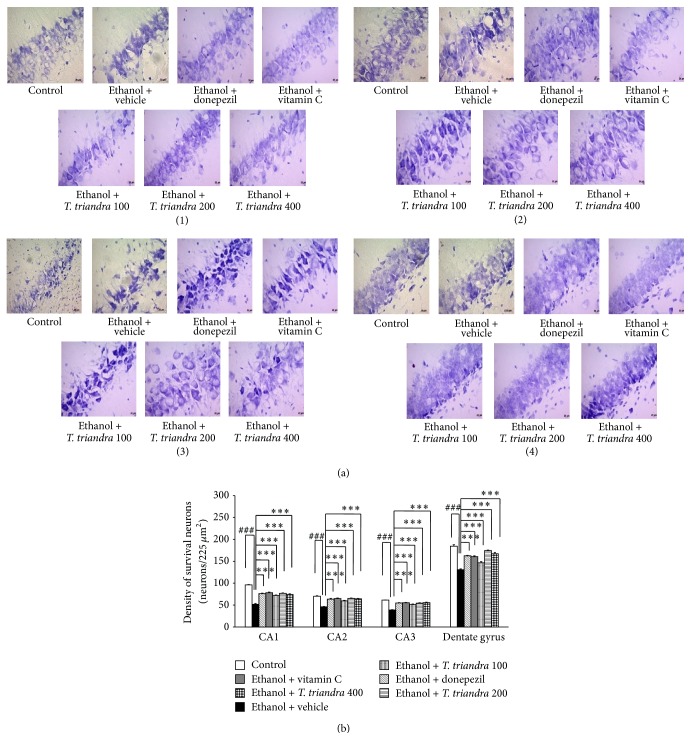
The effect of* T. triandra* extract on the neuron density in CA1, CA2, CA3, and dentate gyrus of hippocampus. (a) Photographs showing the density of the neurons stained with cresyl violet; (b) the bar graph showing density of neurons in various subregions of hippocampus. Data were presented as mean ± SEM, *n* = 6/group. ^###^ *P* value < 0.001 compared with control treated group. ^∗∗∗^ *P* value < 0.001 compared with ethanol dependence treated group which received vehicle.

Effect of* T. triandra* on neuron density in various subregions of hippocampus including CA1, CA2, CA3, and dentate gyrus was shown in Figures 5(a) and 5(b). Repetitive consumption of alcohol significantly decreased neuron density in CA1, CA2, CA3, and dentate gyrus of hippocampus (*P* value < 0.001 all, compared with control rats). Ethanol dependence rats which received either donepezil or vitamin C significantly attenuated the decreased neuron density induced by alcohol in all subregions mentioned earlier of hippocampus (*P* value < 0.01 all, compared with ethanol dependence rats which received vehicle). Interestingly, all doses of* T. triandra* extract treatment significantly counteracted the decreased neuron density induced by alcohol consumption in hippocampus (*P* value < 0.01 all, compared with ethanol dependence rats which received vehicle).

### 3.5. Effect of* T. triandra* on Oxidative Stress Markers

Table 2 demonstrated the effects of* T. triandra* on oxidative stress markers including malondialdehyde (MDA) level and the activities of main scavenger enzymes such as superoxide dismutase (SOD), catalase (CAT), and glutathione peroxidase (GSH-Px) in hippocampus. The current data showed that alcohol consumption significantly enhanced MDA level (*P* value < 0.001, compared with control rats) but decreased SOD, CAT, and GSH-Px (*P* value < 0.001, 0.01, and 0.01, resp., compared with control rats) enzyme activities. Treatments with donepezil, vitamin C, and* T. triandra* at doses of 100, 200, and 400 mg·kg^−1^BW produced the significant mitigation effect on the elevation of MDA level (*P* value < 0.01 all, compared with alcoholic rats which received vehicle) in hippocampus of ethanol dependence rats. The significant attenuation of SOD activity induced by ethanol in hippocampus was also observed in ethanol dependence rats which received donepezil or vitamin C or* T. triandra* at doses of 100, 200, and 400 mg·kg^−1^BW (*P* value < 0.001, 0.001, 0.01, 0.001, and 0.01, resp., compared with ethanol dependence rats which received vehicle). In addition, ethanol dependence rats treated with donepezil showed a significant attenuation effect on the decreased CAT and GSH-Px activities induced by alcohol (*P* value < 0.01, all, compared with ethanol dependence rats which received vehicle) whereas those which received vitamin C showed only the significant change of CAT activity in hippocampus (*P* value < 0.05, compared with ethanol dependence rats which received vehicle). It was found that* T. triandra* extract at doses of 100, 200, and 400 mg·kg^−1^BW significantly mitigated the enhanced MDA level induced by ethanol consumption (*P* value < 0.001 all, compared with ethanol dependence rats which received vehicle). The significant increased SOD activity in hippocampus was also observed in alcoholic rats which were treated with extract at all doses used in this study (*P* value < 0.01, 0.001, and 0.01, resp., compared with ethanol dependence rats which received vehicle). However, the significant increased CAT activity in hippocampus was observed only in ethanol dependence rats which were treated with low and medium doses of extract (*P* value < 0.01 all, compared with ethanol dependence rats which received vehicle). Treatment with* T. triandra* at the dosage range used in this study also mitigated the decrease of GSH-Px activity in hippocampus of ethanol dependence rats (*P* value < 0.01, 0.001, and 0.01, resp., compared with ethanol dependence rats which received vehicle).

## 4. Discussion

The data obtained from this study demonstrated that the ethanol dependence rats used in this study were hyperactive and increased responsiveness to environmental stimuli, such as startle, increased puffing and hair ruffling, irritability, and hypervigilance similar to that observed in the previous study [17]. The average ethanol concentration in the blood of all ethanol dependence rats prior to the intervention was 9.89 ± 0.86 mg/dL. According to this model, it was found that no serious signs of physical dependence such as spasticity and convulsion were observed. Therefore, the animal model used in this study successfully induced mild to moderate ethanol dependence.

It was found that ethanol dependence rats induced by repetitive consumption of alcohol showed the increased escape latency but decreased retention time together with the neurodegeneration of hippocampus and hypocholinergic function. This was in agreement with the previous studies [4–8, 26, 27]. It has been reported that chronic alcohol consumption induces hippocampal damage, spatial memory impairment [28], together with the decreased oxidative stress [19]. Therefore, the results obtained from this study suggested that the elevation of oxidative stress reflected by the increased MDA level in hippocampus was responsible for the hippocampal damage and memory impairment. In addition, the decreased cholinergic function induced by chronic ethanol consumption also plays the important role in the improved hippocampal damage and memory impairment. It was found that donepezil and vitamin C could attenuate an enhanced oxidative stress and a cholinergic impairment which in turn improved memory deficit [10, 29, 30]. In addition,* T. triandra* leaves extract at all doses used in this study could also mitigate both the enhanced escape latency and the decreased retention time. Moreover, it also increased neuron density and improved oxidative stress status and cholinergic function in hippocampus of ethanol dependence rats. Previous studies clearly demonstrated that the neurons in hippocampus play the crucial role in the spatial memory [31]. Therefore, we did suggest that the improved oxidative stress which in turn increased neuron density in hippocampus was responsible for the improved memory impairment in ethanol dependence rats which received* T. triandra* leaves extract. In addition, the improved cholinergic function also played a role in the improved memory deficit in ethanol dependence rats which received a medium dose of* T. triandra* leaves extract. It was found that high dose of the extract failed to show the suppression of AChE in hippocampus. The possible explanation might be associated with the masking effect of the other ingredients in the crude extract of* T. triandra* leaves. Since* T. triandra* contained high concentration of polyphenolic compounds and high concentration of quercetin and these compounds also exerted neuroprotective effect against various insults [32–36], we did suggest that the neuroprotective effect of* T. triandra* extract observed in this study might be partly associated with quercetin. However, this still required further investigation.

## 5. Conclusions

The current study clearly demonstrates that* Tiliacora triandra* provides neuroprotective and cognitive enhancing effects in ethanol dependence rats. Therefore, it should be used as functional food and nutraceutical product to improve memory impairment and brain damage induced by ethanol dependence. The possible mechanism of extract may be due to the antioxidant and the suppression of acetylcholinesterase activity in hippocampus. Further studies are required to investigate the possible active ingredients, detail mechanism, and signal pathways.

## Figures and Tables

**Table 1:** Gradient program of HPLC analysis.

Times (minutes)	Solvents (%)	
	A (methanol)	B (2.5% acetic acid)
0	10	90
17	70	30
18	100	—
20	100	—
20.5	10	90
25	10	90

**Table 2:** The effect of *T. triandra* extract on oxidative stress markers including malondialdehyde (MDA) level and the activities of superoxide dismutase (SOD), catalase (CAT), and glutathione peroxidase (GSH-Px) in hippocampus.

Groups/oxidative stress markers	MDA (nmol/mg·protein)	SOD (U/mg·protein)	CAT (U/mg·protein)	GSH-Px (U/mg·protein)
Control	0.0017 ± 0.0002^***^	2.80 ± 0.39^***^	12.38 ± 0.61^**^	3.473 ± 0.43^**^
Ethanol + vehicle	0.012 ± 0.0007^###^	0.98 ± 0.31^###^	8.65 ± 1.73^##^	2.473 ± 0.18^##^
Ethanol + donepezil	0.005 ± 0.0003^***^	2.80 ± 0.39^***^	13.18 ± 1.95^**^	4.620 ± 0.61^**^
Ethanol + vitamin C	0.004 ± 0.0007^***^	2.80 ± 0.39^***^	10.91 ± 0.64^*^	3.013 ± 0.10
Ethanol + *T. triandra* 100	0.005 ± 0.0005^***^	1.62 ± 0.43^**^	12.877 ± 0.72^**^	4.30 ± 0.53^**^
Ethanol + *T. triandra* 200	0.004 ± 0.0005^***^	2.12 ± 0.27^***^	11.55 ± 1.09^**^	6.29 ± 0.52^***^
Ethanol + *T. triandra* 400	0.004 ± 0.0004^***^	1.56 ± 0.18^**^	9.31 ± 1.63	3.92 ± 0.56^**^

Values are expressed as means ± SEM from 6 animals in each group.

^##^ *P* value < 0.01 and ^###^ *P* value < 0.001 compared with control treated group.

^*^ *P* value < 0.05, ^**^ *P* value < 0.01, and ^***^ *P* value < 0.001 compared with ethanol dependence rats which received vehicle.
